# Quantitative analysis of human ankle characteristics at different gait phases and speeds for utilizing in ankle-foot prosthetic design

**DOI:** 10.1186/1475-925X-13-19

**Published:** 2014-02-26

**Authors:** Zahra Safaeepour, Ali Esteki, Farhad Tabatabai Ghomshe, Noor Azuan Abu Osman

**Affiliations:** 1 Department of Prosthetics and Orthotics, University of Social Welfare and Rehabilitation Sciences, Tehran, Iran; 2 Department of Biomedical Engineering and Physics, Shahid Beheshti University of Medical Sciences, Tehran, Iran; 3 Department of Ergonomics, University of Social Welfare and Rehabilitation Sciences, Tehran, Iran; 4 Department of Biomedical Engineering, Faculty of Engineering, University of Malaya, Kuala Lumpur, Malaysia

**Keywords:** Ankle biomechanics, Walking, Prosthesis, Power, Ankle-foot mechanisms, Hysteresis, Quasi-stiffness

## Abstract

**Background:**

Ankle characteristics vary in terms of gait phase and speed change. This study aimed to quantify the components of ankle characteristics, including quasi-stiffness and work in different gait phases and at various speeds.

**Methods:**

The kinetic and kinematic data of 20 healthy participants were collected during normal gait at four speeds. Stance moment-angle curves were divided into three sub-phases including controlled plantarflexion, controlled dorsiflexion and powered plantarflexion. The slope of the moment-angle curves was quantified as quasi-stiffness. The area under the curves was defined as work.

**Results:**

The lowest quasi-stiffness was observed in the controlled plantarflexion. The fitted line to moment-angle curves showed *R*^2^ > 0.8 at controlled dorsiflexion and powered plantarflexion. Quasi-stiffness was significantly different at different speeds (*P* = 0.00). In the controlled dorsiflexion, the ankle absorbed energy; by comparison, energy was generated in the powered plantarflexion. A negative work value was recorded at slower speeds and a positive value was observed at faster speeds. Ankle peak powers were increased with walking speed (*P =* 0.00).

**Conclusions:**

Our findings suggested that the quasi-stiffness and work of the ankle joint can be regulated at different phases and speeds. These findings may be clinically applicable in the design and development of ankle prosthetic devices that can naturally replicate human walking at various gait speeds.

## Background

The overall function of a human ankle during the stance phase of walking can be explored in terms of moment versus angle relation [1]. This relation reveals an almost linear loop-shaped curve for the majority of stance phase at slow and normal walking speeds [1-4]. The curve shows a clockwise hysteresis loop at slower speeds, whereas a counter-clockwise loop is displayed at higher speeds [5]. Considering the moment-angle curve, researchers suggested that human ankle can be replaced with a passive rotational spring-damper system at slow and normal speeds, but an augmented active mechanism is necessary at fast speeds [3,5,6].

The conventional passive prosthetic feet are limited to relatively rigid ankle mechanisms. This condition has prompted researchers to develop passive elastic prosthetic feet with energy storing and returning capabilities [1]. Although energy-storing prostheses exhibit more advantages than conventional feet [7], both devices show the same biomechanical behaviors which deviate from normal ankle function [1,5,8]. These limitations can be decreased by designing the mechanical parameters of the prosthetic ankle based on the human ankle characteristics [9-11].

Human ankle characteristics, such as quasi-stiffness and work derived from the moment-angle curve, are keys to design the prosthetic feet [1,3,5,6,12]. Quasi-stiffness refers to the slope of the curve and defined as overall ankle resistance to motion [3,13-15]. Previous studies indicated that ankle quasi-stiffness is an important design aspect which should be tuned based on gait sub-phases and speeds in a successful prosthetic design. The values of ankle quasi-stiffness can be used as a guideline for researchers to select and adjust the spring coefficient of the prostheses [3,5,16]. Ankle work is calculated from the area under the curve and quantifies the amount of energy absorbed or produced by the joint structures [2,5]. In addition, the ankle displays negative work in mid-stance and positive work at terminal stance. The ankle work and quasi-stiffness varies throughout a gait cycle or in terms of gait speed [2,5,8,17]. A successful prosthetic design should incorporate adaptive characteristics similar to those of the human ankle in a gait cycle or at different speeds [1,3,5,6].

Previous studies explored the ankle moment-angle relation at different speeds and showed some aspects of ankle function [3,5,15,18]. However, the findings of these studies cannot be directly applied in the design of ankle prostheses because a range of ankle characteristics at different gait phases and speeds have not been presented.

In this study, the ankle moment-angle curve was quantitatively analyzed using a motion capture system. Useful information in terms of quasi-stiffness and work was also analyzed and presented in distinct sub-phases of stance at a range of walking speeds that can be applied for prosthetic designs.

## Methods

### Participants and protocol

Twenty healthy volunteers, 14 males and 6 females, without history of foot or ankle and lower extremity orthopedic or neurological pathologies participated in this study. On average, the participants were 23.4 (SD 3.7) years old, 67.6 (SD 10.2) kg in weight, and 174.2 (SD 7.7) cm in height. All of the participants provided written informed consent approved by the Ethics Committee of the University of Social Welfare and Rehabilitation Sciences.

Analyses were performed in a standard gait laboratory which is equipped with five infrared cameras (Vicon 460, Vicon Motion System Ltd., UK) and two force plates (Kistler Instrument AG, Switzerland). Data were collected at a rate of 100 Hz. Reflective markers were placed on the anatomical landmarks according to the recommendation of Vicon ‘Plug-In-Gait’ marker set [19]. This set included the ankle (lateral malleolus), toe (dorsum of the foot between first and second metatarsals), heel, tibia (one third distal), knee (lateral femoral condoyle), femur (one third distal) and ASIS (anterior superior iliac spines). A marker was placed on the sacrum to compute the average walking speed [5]. Each participant was instructed to walk barefoot at four walking speeds including normal, slow, very slow and fast speeds. At first, they were instructed to walk at their self-selected normal speed. Then they were asked to walk slower and faster than their normal speeds. The trials were accepted when the foot was completely in contact with the platform [6].

### Data processing

The marker positions and ground reaction forces were processed using the Vicon Plug-In-Gait model (Workstation version 4.6) to derive the ankle angle (degree), moment (N m), and power (W). Ankle moments were computed by an inverse dynamics approach. Ankle power was defined as multiplying the ankle moment by ankle angular velocity. Moment and power were then normalized by each individual’s body mass (N m kg^−1^, W kg^−1^). Average walking speed was defined as the total displacement of the sacrum marker divided by time at a defined distance [5]. Kinetic and kinematic data were low-pass filtered using a zero-lag, sixth-order Butterworth filter with a cut-off frequency of 10 Hz [20]. Linear interpolation was applied to the data points to establish equal lengths of data sets [2]. Zero ankle position was defined as the point where the foot segment was perpendicular to the tibia segment. The dorsiflexion angle and moment of the ankle were considered positive.

**Figure 1:**
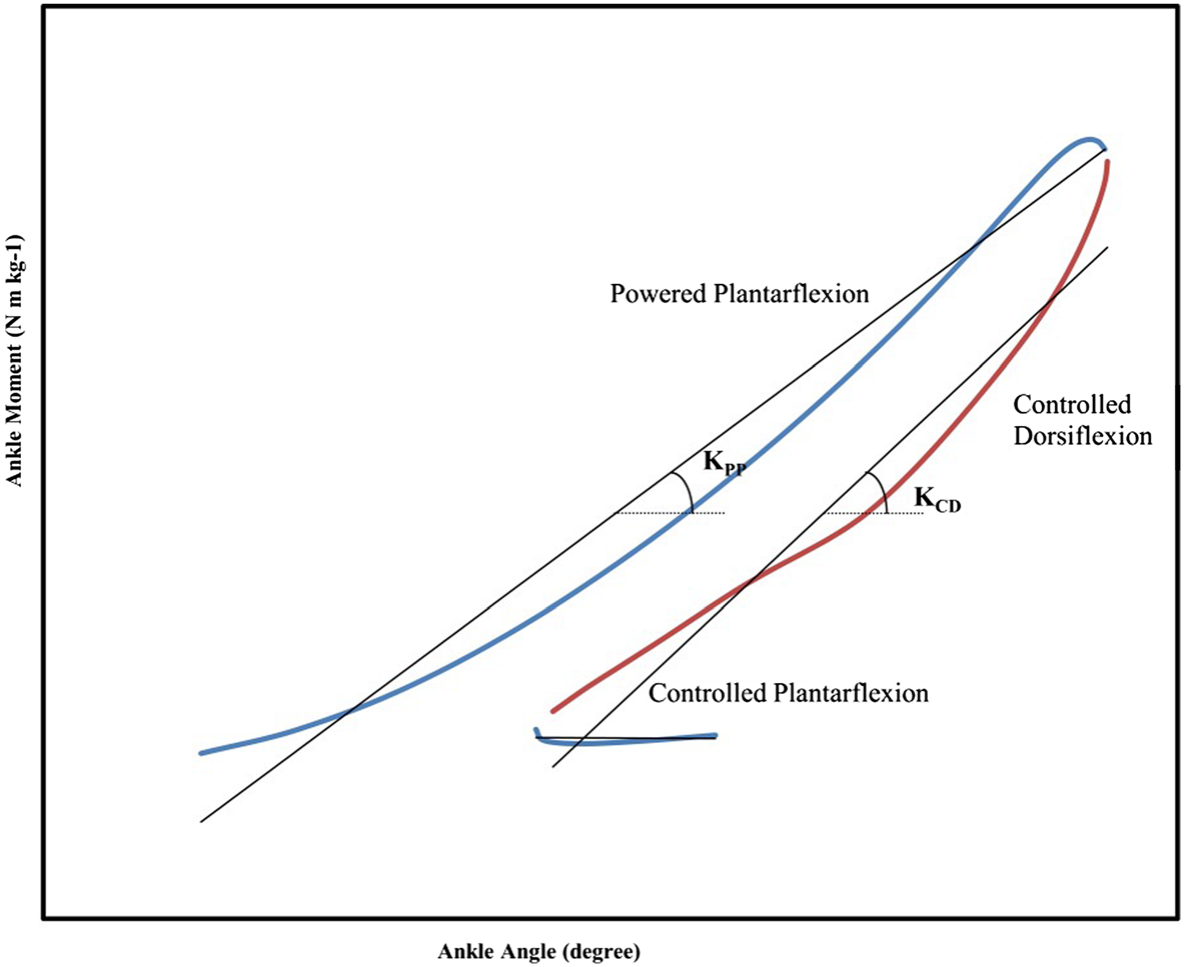
Typical ankle moment versus angle loop in the stance phase of walking was divided into three sub-phases: controlled plantarflexion, controlled dorsiflexion, and powered plantar flexion.

Each moment-angle loop in the stance phase was divided into three sub-phases: controlled plantarflexion, controlled dorsiflexion; and powered plantarflexion [12,21]. The beginning of the controlled plantarflexion phase was characterized by a heel strike and ended at the maximum plantarflexion in the early stance. The subsequent controlled dorsiflexion phase was identified from the end of controlled plantarflexion to the maximum ankle dorsiflexion in mid-stance, in which the ankle angle increased and power remained negative. Powered plantarflexion was defined as the phase from the end of controlled dorsiflexion when ankle power changed to positive values to the toe-off. In this sub-phase, the ankle obtained the maximum plantarflexion and power remained positive. The quasi-stiffnesses or K of the ankle (N m kg^−1^ rad^−1^) in controlled plantarflexion (K_CP_), controlled dorsiflexion (K_CD_), and powered plantarflexion (K_PP_) were estimated as the slopes of the corresponding linear regression lines to the moment-angle data at each sub-phase (Equation 1) (Figure 1) [1-3,14,15,18].

(1) $$K=\frac{\mathrm{dm}}{\mathrm{d\theta}}$$

Where M is the ankle moment, θ is the joint angle and K stands for the quasi-stiffness.

The values of work exerted by the ankle (N m rad kg^−1^) in controlled plantarflexion (W_CP_), controlled dorsiflexion (W_CD_), and powered plantarflexion (W_PP_) were calculated as the area under moment-angle curve at each sub-phase on the basis of the trapezoidal approximation approach. The area within the loop was considered as the total work exerted by the ankle in the stance phase (W_total_); this area was computed by subtracting the absolute areas under powered plantarflexion (|W_PP_|) and controlled dorsiflexion (|W_CD_|) curves [2,3,5].

### Statistical analysis

The normal distribution of data was examined by Kolmogorov-Smirnov test. Linear regression models using least squares approach were applied to examine the relationship between moment and angle. The goodness of fit of the regression models was evaluated using the coefficients of determination (*R*^2^). Repeated measures ANOVA with Bonferroni-adjusted post-hoc test was conducted to compare the means at different sub-phases and speeds. Statistical analyses were performed using SPSS version 19 (SPSS Inc., Chicago, IL, USA). *P* < 0.05 was considered as the significance level of all of the tests.

## Results

The average walking speeds at the four categories were 0.95 (0.07), 1.06 (SD 0.09), 1.25 (SD 0.13), and 1.58 (SD 0.12) m s^−1^. The mean differences among the walking speeds in the four categories were statistically significant (*P* < 0.05).

The regression lines fitted to the controlled plantarflexion moment-angle data showed 0.49 ≤ *R*^2^ ≤ 0.75 (Table 1). No significant differences were found between K_CP_s at all of the speeds used in this study (*P* > 0.05)*.* Moment curves versus angle curves in the controlled dorsiflexion phase were observed in the regression models, but *R*^2^ decreased as speed increased (0.83 ≤ *R*^2^ ≤ 0.96, Table 1). Statistically significant differences were found in K_CD_ in terms of walking speeds. K_CD_ at fast and normal speeds was significantly higher than that at slow and very slow speeds (*P* = 0.00, Table 1)*.* The moment-angle curves of powered plantarflexion showed *R*^2^ > 0.95 (Table 1). The results showed no significant differences between K_PP_s at all of the walking speeds (Table 1).

**Table 1:** **Mean (SD) of the ankle quasi-stiffness (N m kg**^**−1**^ **rad**^**−1**^**) and *R***^**2 **^**at four walking speeds**

	**Very slow speed**	**Slow speed**	**Normal speed**	**Fast speed**
K_CP_	−0.63 (SD 0.93)	−0.44 (SD 1.03)	−0.20 (SD 0.90)	−0.43 (SD 0.76)
K_CD_	4.57 (SD 0.77)^a,b^	4.47 (SD 0.62)^a,b^	5.47 (SD 0.76)^a^	5.80 (SD 1.05)^b^
K_PP_	4.78 (SD 0.62)	4.46 (SD 0.76)	4.58 (SD 0.71)	4.53 (SD 0.85)
R^2^_CP_	0.75 (SD 0.35)	0.70 (SD 0.32)	0.58 (SD 0.29)	0.49 (SD 0.35)
R^2^_CD_	0.96 (SD 0.04)	0.95 (SD 0.03)	0.91 (SD 0.06)	0.83 (SD 0.09)
R^2^_PP_	0.95 (SD 0.03)	0.97 (SD 0.03)	0.98 (SD 0.01)	0.98 (SD 0.01)

^a,b^show the significant differences between K_CD_ at fast and normal speeds and K_CD_ at very slow and slow walking speeds (*P* < 0.05).

**Figure 2:**
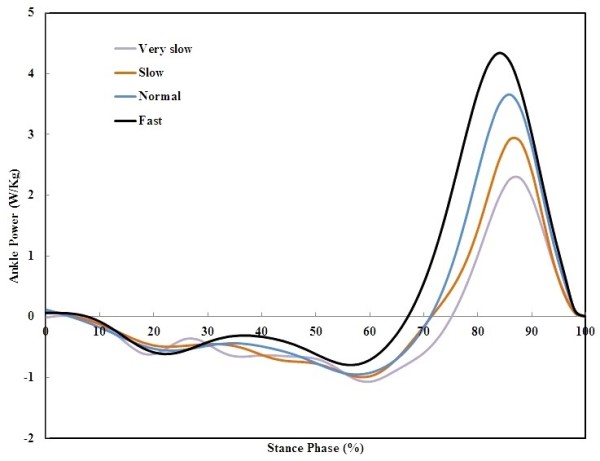
Ankle power versus stance time at four walking speeds.

**Figure 3:**
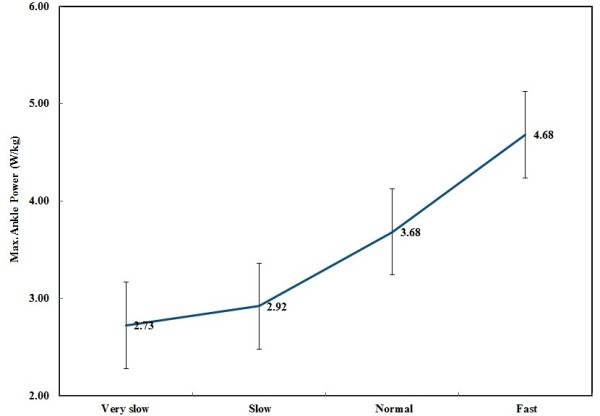
Ankle Max power at four walking speeds.

Statistically significant differences were found in W_total_ in all speeds (*P* = 0.00, Table 2). Moreover, in early stance (controlled plantarflexion phase) and mid-stance to terminal stance (controlled dorsiflexion phase), power was negative, indicating energy absorption. In the pre-swing (powered plantarflexion) phase, power was positive, showing energy generation in this period (Figure 2). The mean ± SD of stance power at slow to fast speeds were −0.12 (0.13), 0.03 (0.15), 0.17 (0.18), 0.46 (0.22) W kg^−1^. The mean power differences were significant (*P* < 0.05). Maximum ankle power was increased with walking speed (Figure 3).

**Table 2:** **Mean (SD) of the ankle work (N m rad kg**^**−1**^**) at four walking speeds**

	**Very slow speed**	**Slow speed**	**Normal speed**	**Fast speed**
│W_CP_│	0.01 (SD 0.00)	0.01 (SD 0.00)	0.01 (SD 0.00)	0.01 (SD 0.00)
│W_CD_│	0.33 (SD 0.07)	0.35 (SD 0.09)	0.25 (SD 0.05)	0.20 (SD 0.05)
│W_PP_│	0.28 (0.07)	0.40 (0.09)	0.41 (0.07)	0.55 (SD 0.1)
W_total_	−0.05 (0.03)^a^	0.05 (0.03)^a^	0.15 (0.05)^a^	0.35 (SD 0.08)^a^

^a^shows the significant differences between W_total_ at all speeds (*P* < 0.05).

## Discussion

In the present study, a range of ankle quasi-stiffness and work was investigated in terms of three distinct stance sub-phases at four walking speeds. This data set can be beneficial for designers who define the mechanical requirements of prosthetic ankle-foot devices.

Findings showed that the general pattern of ankle behavior were consistent with previous studies, demonstrating that human ankle changes from a passive to an active system in response to increased walking speed [5,18,22].

In the controlled plantarflexion phase, findings showed lower values of quasi-stiffness and work than other sub-phases. In this period, ankle begins to plantarflex under the eccentric contraction of dorsiflexor muscles to provide shock absorption, and to control the rate of floor impact [21]. Regression lines fitted to the moment versus angle data showed a great variability in this period. This might be due to the number of the kinetics and kinematics data in the controlled plantarflexion (about 5 data points at the frequency of 100 Hz) as this phase occurs in a short period of time. However, fits with high R^2^s values were seen in the majority of the participants. Therefore, spring-like behavior of ankle joint can be considered in the prosthetic design.

In the controlled dorsiflexion phase, ankle showed moment-angle relationship with acceptable R^2^ values. In this interval, tibia moves forward over the stationary foot, ankle begins and continues to dorsiflex with increasing plantarflexor moment that peaks at the end of the phase [21]. Moreover, negative work is done by ankle plantarflexors to decelerate the rate of leg forward movement [3,18,21]. Thus, it could be simply assumed that ankle shows spring-like behavior with considerable amount of energy absorption in the controlled dorsiflexion phase. Considering the walking speed, results indicated that ankle becomes stiffer at higher speeds in the controlled dorsiflexion. Similarly, Shamaei et al. [3] concluded that movement of the ankle joint becomes more nonlinear in controlled dorsiflexion phase at higher speeds. It can be inferred that ankle acts possibly more linearly elastic in slower speeds, like a linear spring, but at higher speeds more complicated and augmented systems are added. Thus, a spring with an adjustable stiffness modulated according to the walking speed can be considered in the prosthetic design in this period.

During the powered plantarflexion interval, body weight abruptly transfers to the contra lateral limb. Ankle joint plantarflexes under a declining moment while plantarflexors produces a positive peak power to push the body forward [21]. The results at the powered plantarflexion phase showed positive peak power at all speeds that were increased with walking speed. Hence, results suggest that in powered plantarflexion interval, ankle functions like a spring–damper structure at very slow speed. However, the damping effect gradually vanishes at slow speeds. At higher walking speeds, an active source of energy progressively turns up. This suggests the demands for implementation of augmented systems (such as motor) in combination with passive spring-damper elements at fast walking speeds.

This study was performed on 20 healthy young participants at different ranges of age, mobility, and walking speed. Therefore, the results of the analyses cannot be applied in other populations, such as those with lower limb amputees or diabetic patients. The kinetic and kinematic data of the ankle were derived using a single-segment Vicon Plug-In-Gait model; as such, this factor should be considered when our results are applied since power and work may be overestimated [23].

## Conclusions

This work provides a quantitative understanding of ankle characteristics aimed to be assistive for designers in developing new ankle-foot prostheses. The values of the ankle quasi-stiffness and work presented in this study can be used as a guideline for the selection and adjustment of the spring coefficient of the prostheses. However, variations in the parameters exist at different sub-phases and walking speeds. These adjustment strategies should be considered in the design of ankle prosthetic systems to attain more natural behavior.

## Abbreviations

KCP: Quasi-stiffness of the ankle in controlled plantarflexion; KCD: Quasi-stiffness of the ankle in controlled dorsiflexion; KPP: Quasi-stiffness of the ankle in powered plantarflexion; WCP: Work exerted by the ankle in controlled plantarflexion; WCD: Work exerted by the ankle in controlled dorsiflexion; WPP: Work exerted by the ankle in powered plantarflexion.

## Competing interests

The authors declare no competing interests.

## Authors’ contributions

ZS carried out the research, participated in the study design, data analysis and drafted the manuscript. AE and FTG involved in study design, revising the manuscript and analysis of data. NAAO revised it critically for important intellectual content. All authors read and approved the final manuscript.
